# Open-Hybrid Aortic Stent Placement for Recurrent Coarctation in Complex Single Ventricles

**DOI:** 10.1016/j.atssr.2024.09.021

**Published:** 2024-10-16

**Authors:** Andrew K. Morse, Blaz Podgorsek, Julija Dobrila, Zachary A. Cerra, Kiran K. Mallula, Muhammad S. Khan, Christopher E. Greenleaf, Jorge D. Salazar, Damien J. LaPar, Peter C. Chen

**Affiliations:** 1 Children’s Heart Institute, Children’s Memorial Hermann Hospital, Division of Pediatric & Congenital Heart Surgery, University of Texas Health Science Center at Houston McGovern Medical School, Houston, Texas; 2 Children’s Heart Institute, Children’s Memorial Hermann Hospital, Division of Pediatric Cardiology, University of Texas Health Science Center at Houston McGovern Medical School, Houston, Texas

## Abstract

Recurrent coarctation of the aorta in patients with hypoplastic left heart syndrome requires timely intervention to limit ventricular dysfunction and atrioventricular valve regurgitation. Current strategies include catheter-based intervention in adequately sized patients or surgical arch augmentation at the time of a concomitant operation. We report an open-hybrid surgical technique with placement of a balloon-expandable stent that can later be expanded to an adult size as the patient grows. Limiting the arch dissection reduces the risk to the left recurrent laryngeal nerve and shortens anterograde cerebral perfusion time.

Recurrent coarctation of the aorta after the Norwood operation for hypoplastic left heart syndrome (HLHS) occurs in ∼10% to 40% of patients in recent studies and has been shown to be a risk factor for long-term morbidity and mortality in single-ventricle patients.^1^^,^^2^ The current management strategies include percutaneous stenting or open surgical repair, each of which has limitations that prevent them from being the technique of choice.^3^^,^^4^

We present an open-hybrid stenting technique to address recurrent coarctation in patients too small to receive an adult-sized stent at the time of cardiac catheterization. This technique limits morbidity associated with an open surgical approach and allows for future ballooning of the stent to an adult size as the patient grows.

## Technique

After redo sternotomy, a dissection of the cardiac mass and great vessels is completed, including the head and neck vessels. Limited dissection of the aortic arch can be performed, but enough dissection is necessary to allow for a longitudinal incision along the proximal transverse arch for stent placement. Arterial cannulation of the innominate artery allows for aortic cross-clamp placement across the base of the head and neck vessels during antegrade cerebral perfusion (ACP). When performed during the Glenn procedure, right atrial and superior vena cava cannulation is then performed, and cardiopulmonary bypass (CPB) is commenced after adequate heparinization has been confirmed. The patient is cooled to 25 °C, and the cross-clamp is applied, followed by delivery of antegrade del Nido cardioplegia.

**Figure 1:**
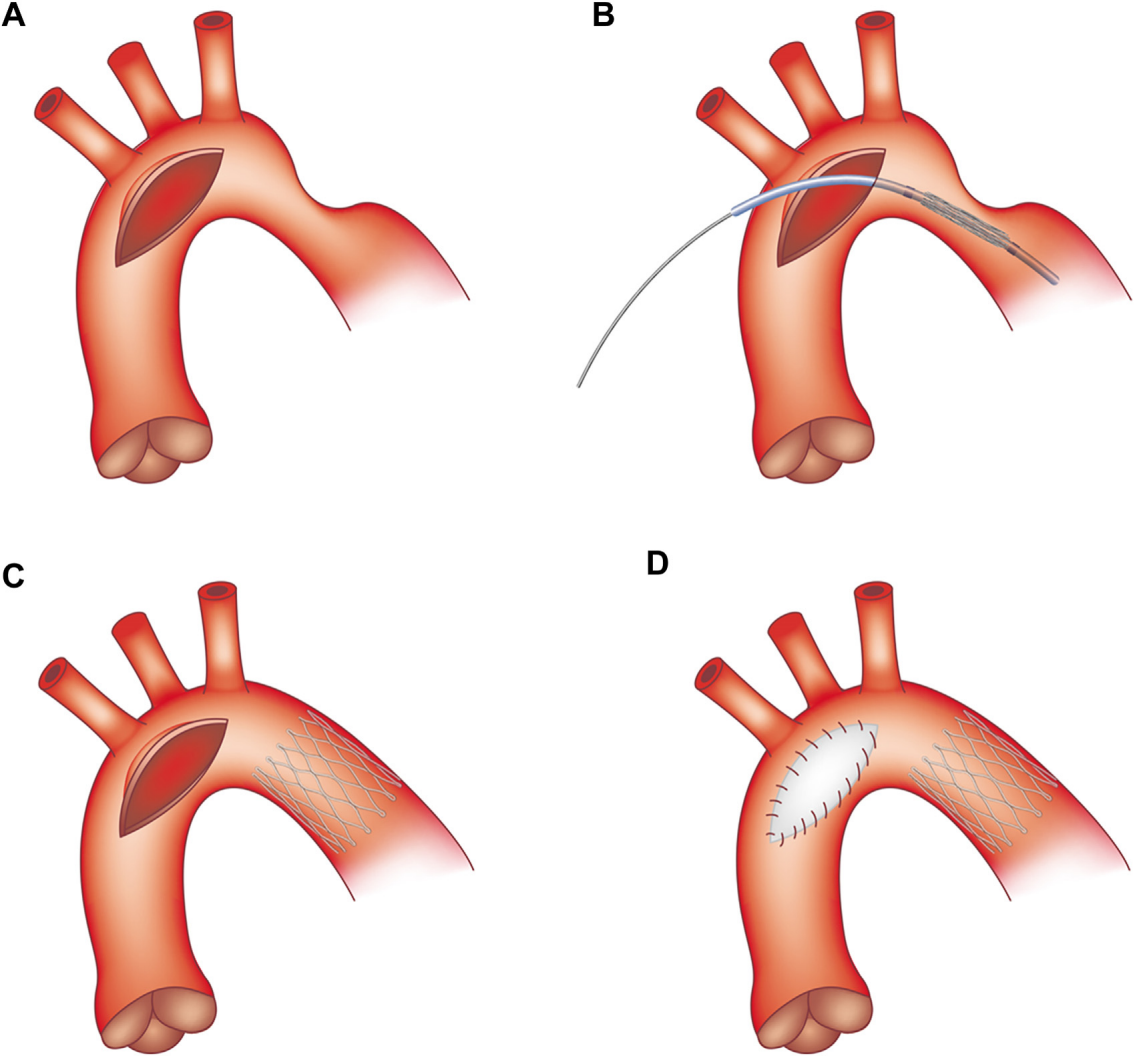
Diagram of operative steps for open-hybrid stenting: (A) Longitudinal aortic incision is made proximal to the site of recurrent coarctation, (B) direct insertion of wire and stent with balloon dilation catheter under direct vision, (C) ballooning of the stent to the desired size; and (D) aortotomy patch closure and aortic arch augmentation.

Under cardioplegic arrest, the aortic cross-clamp is repositioned to the proximal ascending aorta, and ACP is initiated with snaring of the head and neck vessels. The aortic arch is opened longitudinally proximal to the site of recurrent arch obstruction, and the area of stenosis is identified (Figure 1, Video). Guidewire access using a J-tip, ultrastiff, 0.035-inch wire is obtained under direct vision, and the appropriate adult-sized vascular stent is then advanced into position across the coarctation segment. The stent is presized based on a preoperative angiogram or computed tomographic images, typically 1- to 2-mm greater than the proximal vessel caliber.

**Figure 2:**
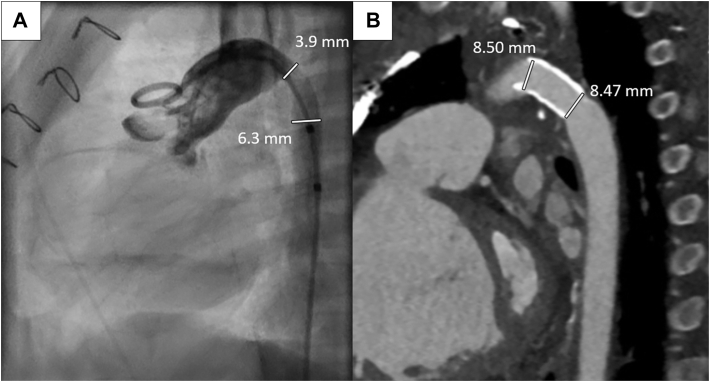
(A) Preoperative angiogram demonstrates the area of recurrent coarctation and (B) postoperative computed tomographic image with patent open hybrid stent.

The stent is prepared by placing a gentle curve along the stent before placement of the aortic cross-clamp. When adequately positioned, the balloon is inflated to the desired size, and the guidewire is removed. If necessary, additional stents can be placed, when necessary, for a long-segment stenosis. An aortotomy patch is then placed to further augment the arch and prevent future stenosis at the site. A concomitant Glenn procedure can be performed once the cross-clamp is removed while the patient is rewarming. Postoperative computed tomography demonstrates improved arch diameter with stent patency (Figure 2).

## Comment

Recurrent coarctation occurs in 11% to 37% of patients after the Norwood operation, leading to increased afterload on the single ventricle and resulting in depressed ventricular function and worsening atrioventricular valve regurgitation, ultimately leading to long-term morbidity and mortality.^1^ The current management strategy of recurrent aortic arch obstruction requires surgical or percutaneous intervention to address the site of stenosis.

When reintervention is required in smaller patients, vascular access can be a limiting factor in the ability to place an adequately sized stent that can eventually be dilated to an adult size or fractured and restented with an adult-sized stent.^5^ Although balloon angioplasty is often considered a first-line option, this technique can be ineffective in up to 20% of patients due to vessel recoil, vessel torsion, or external compression.^3^ Surgical arch augmentation of the stenotic segment typically is performed concomitantly at the time of the Glenn operation with durable long-term results.^4^ However, dissection of the distal arch to provide a suitable segment of native aorta distal to the coarctation segment can be challenging and places the left recurrent laryngeal nerve at risk for traction or cautery injury.^6^ Additionally, patch augmentation of the stenotic segment will require a significant duration of antegrade cerebral perfusion (ACP).

Open-hybrid aortic stenting provides a surgical alternative that can be performed at the time of the Glenn operation and allows the opportunity to place a stent that can be dilated to an adult size or fractured or restented in the future to an adult size. This technique limits dissection of the distal aortic arch and therefore provides less risk of injury to the left recurrent laryngeal nerve. ACP time is also shortened significantly with this procedure compared with traditional arch augmentation.

The balloon-expandable vascular stents used in the open-hybrid procedure are uncoated, open-cell, premounted, stainless steel stents that allow for percutaneous balloon expansion in the future as the patient ages.^7^ These balloon-expandable devices have previously demonstrated utility in a variety of applications in the field of congenital heart surgery, including intraoperative stenting of branch pulmonary artery stenosis and patent ductus arteriosus stenting in the hybrid management of hypoplastic left heart syndrome.^8^ The use of these devices in the management of recurrent coarctation is advantageous in the neonatal population because it allows for expansion of the stent to meet the needs of the growing child, making it a more appealing palliation strategy than a traditional fixed-size stent. In addition to the benefit of a longer duration palliation, the open-hybrid procedure also reduces the duration of ACP time and the potential risk to the recurrent laryngeal nerve with arch augmentation.

At our center, open-hybrid stenting of recurrent coarctation after the Norwood operation has been performed with promising short-term outcomes in patients too small for placement of a stent that can be ballooned to an adult size in the future at the time of their pre-Glenn cardiac catheterization. In this instance, we would advocate for either balloon angioplasty for a short duration before the Glenn operation if the patient is too young or small, or perform an early Glenn with open-hybrid stenting. If the patient’s femoral vessels can accommodate the appropriately sized stent, then this will be performed before any surgical intervention. In conclusion, this technique provides a novel use of balloon-expandable stents as an alternative treatment option for patients with recurrent coarctation.
